# A Novel ‘Train the Trainer’ Emergency Medicine Resident Point-of-Care Ultrasound Course: A Feasibility Study

**DOI:** 10.51894/001c.11650

**Published:** 2020-01-30

**Authors:** Nikolai Butki, Jereme Long, Andrew Butki, William Corser

**Affiliations:** 1 Emergency Medicine Michigan State University/McLaren Oakland; 2 Emergency Medicine Lakeland Health; 3 Emergency Medicine McLaren Oakland; 4 State Wide Campus System MIchigan State University

**Keywords:** ultrasound, train the trainer, pocus

## Abstract

**CONTEXT:**

A novel multi-site ‘train the trainer’ point-of-care ultrasound (POCUS) training course was designed to better meet the graduate medical education learning needs of a geographically dispersed consortium of 16 community-based Michigan emergency medicine (EM) residency programs. The specific aim of this study was to explore the feasibility of using volunteer EM physicians who were novices with ultrasound techniques as instructors for a POCUS course. Additionally, the authors evaluated the effectiveness and consistency of a POCUS course delivered over multiple sites to enhance EM residents’ ultrasound knowledge and skill acquisition.

**METHODS:**

For the initial session, the lead instructor conducted a focused two-hour course with the novice instructors. A subsequent four-hour session was then repeated for EM residents whereby the aforementioned novice instructors provided the hands-on instruction. The residents were given 10-item pre- and 20-item post-course knowledge tests to gauge the effectiveness of the instruction model. After the course, a satisfaction survey was administered to the resident participants and a qualitative open-ended survey to the volunteer EM physicians who served as instructors.

**RESULTS:**

Forty-two EM residents from 11 different residency programs attended at one of the three courses that were offered. After adjustments for size differences in the pre- and post-training tests, 35 (87.5%) of total sample resident learners’ scores proportionately increased from pre- to post-test scores, with five (11.9%) other residents maintaining their pre-course score levels and only two (4.8%) residents experienced a post-score decline. In addition, resident participants responded favorably to a post-course summary evaluation with an average response of 4.8 (0-5 Likert scale) demonstrating overall satisfaction with the course. In the separate qualitative survey given to instructors, comments consistently conveyed a perceived benefit for the volunteer EM physicians.

**CONCLUSIONS:**

The evaluation of this novel model supports the feasibility of the ‘train the trainer’ program. It provides a proof of principle that train the trainer model can be implemented for POCUS training courses. Despite the small sample size, our results show an increase in the pre- to post-test scores among most participating residents. This model provides an additional option for EM residency program educators to consider when developing their POCUS training courses across multiple GME settings.

## INTRODUCTION

Point-of-care ultrasound (POCUS) training is no longer considered a niche within Emergency Medicine (EM), but rather an expected component of Graduate Medical Education (GME) EM training programs.^1–5^ In 2000, POCUS residency training guidelines were released by the American College of Emergency Physicians (ACEP), including a recommendation that residents perform at least 150 procedures to establish minimal competency.^6^ Several articles to date have suggested that POCUS competencies are not being regularly met in many EM residency program settings.^1,3,7–9^

Most POCUS training workshops are one-half to two days in length and follow the traditional model of structured didactic sessions followed by hands-on training with human models or simulation units.^1,3,4^ Some published studies on POCUS education and delivery models have shown positive learner outcomes; however, several have also indicated variable improvement across learners.^1,4,10,11^ A newer model of instruction in GME that has shown promise has been “train the trainer”^12,13^ and “co-learning,”^14,15^ models that involve simultaneous faculty and resident training and “peer training” for senior-junior resident or faculty-faculty peers.^16^

Many residency programs in the authors’ health systems lacked faculty with advanced training to provide advanced POCUS training within their individual programs. In addition, geographic distances had presented barriers to resident participation in courses sponsored by their consortium held at a centralized site. To address these barriers, an innovative POCUS course was designed and delivered utilizing the "train the trainer" model.

### Purpose of study

The purpose of this study was to evaluate the effectiveness and feasibility of using volunteer EM physicians who were less experienced in ultrasound techniques as instructors to enhance EM residents’ POCUS knowledge and skill acquisition. The authors’ overall null hypotheses were that a) participating EM residents would not demonstrate consistent proportionate pre-to-post course US exam score improvements, and b) pre- and post-training score differences would vary significantly across the three training sites. To evaluate the sustainability of this “train the trainer” course, there was also a brief qualitative survey eliciting the volunteer physicians’ perceived benefits of serving as instructors.

## METHODS

An innovative element of this course was the cost-effective use of volunteer EM physicians as instructors for the course. The volunteer instructors were attending physicians or senior residents from the EM residency programs within the consortium. We specifically invited physicians with self-perceived low levels of POCUS competency who had a desire to elevate their own POCUS skills and their ability to teach POCUS.  

The instructors were trained on the morning of the course during a two-hour session. The instructor training consisted of limited lectures and supervised practice obtaining and interpreting images on live models. The three to five instructors for each session shared two live models and two ultrasound machines during the session.

The instructors were incentivized with several benefits for attending this course. They received two hours of close instruction along with supervision of their teaching by a POCUS expert. They had the advantage of hearing the material twice and reinforcing what they learned by teaching others. The instructors also received two hours of 1-A CME credit for the faculty development training and five hours of 1-A CME credit for teaching residents. In addition, they were provided with attestations for their participation in trauma-specific CME and for the scholarly activity of teaching courses to residents outside of their institution.

### Resident Training

This four-hour course was taught using fifteen-minute lectures alternated with thirty-minute practice sessions. The scans taught included cardiac, pulmonary, inferior vena cava, aorta and rapid ultrasound in shock and hypotension (RUSH). The learning objectives for each lecture were to elevate the EM residents’ ultrasound knowledge and to have them identify ideal images including normal and pathologic findings for each scan. The learning objective for each practice session was to elevate the EM residents’ competency in POCUS image acquisition.

During the practice sessions, residents were randomly assigned to a practice station. Each practice station had a live human model on an exam table, a volunteer instructor and an ultrasound machine. Each practice station had a maximum of four residents. The instructor was present at each practice station to answer questions, assist the residents in acquiring ideal images and facilitate discussion. The lead instructor was available during the practice sessions to supervise the instructors and assist residents or answer questions if the instructors were not able.

The course concluded with a capstone interactive group pathology identification examination reinforcing the material taught (See Appendix 1). The course was designed to attest that participants completed 18 goal-directed focused ultrasound scans on a live human and that the learners achieved a Level 2.5 out of 5 in the Accreditation Council for Graduate Medical Education and the American Board of Emergency Medicine EM Milestone PC:12: Other Diagnostic and Therapeutic Procedures: Goal-directed Focused Ultrasound (Diagnostic/Procedural). “Uses goal-directed focused Ultrasound for the bedside diagnostic evaluation of emergency medical conditions and diagnoses, resuscitation of the acutely ill or injured patient, and procedural guidance.”^17^

The pre- and post-course test questions had been written to reflect the learning objectives for the course. Institutional review board approval was obtained for administration of the tests to willing residents and the data collection process. Hard copy tests were administered prior to the start and immediately after the course.

All hard copy pre- and post-course test forms were collected after the participants completed the exam. Study data was entered into a de-identified password-protected data set using randomly assigned study identification numbers. SPSS version 24 analytic software ^18^ was used to generate a series of descriptive statistics concerning sample respondent characteristics and conduct a series of inferential Wilcoxon Matched Pair Signed Rank t tests comparisons ^19^ to examine for possible significance of proportionate pre-to-post-course test score differences. Finally, a series of two-way Analysis of Covariance (ANCOVA) procedures to control for the possible influence of respondent characteristics (e.g., gender, PGY year) on pre-post score differences.^20^ Before analyses, scores from the more comprehensive post-course test were divided in half to accommodate for the different number of equally-weighted items in the pre- (n = 10 items) versus post-course (n = 20 items) tests.

Before the study, the analyst author (WC) used G*Power 3.10.10 software ^21^ to generate minimal sample size calculations for the primary study endpoint: possible pre-post US exam differences per each resident. This indicated that a final sample size of at least 35 EM residents would afford the authors a 0.80069 one minus β level of statistical power to detect statistically significant *within group* (e.g., pairs of individual residents’ proportionate pre- and post-score differences) observing a two-tailed Alpha of 0.05 p value level of significance. This was also based on both a fairly conservative 0.50 (i.e., *Medium*) Effect Size t for the hypothesized influence of training on pre-post POCUS course score differences as generally reported in Favot, et. al., 2015.^4^

In addition to resident performance the perceived benefits for the volunteer instructors were evaluated with a qualitative survey sent out in an email after the course. The survey asked: Q1. “Please describe your overall faculty experiences during this”train the trainer” ultrasound workshop series. What positive or negative aspects of this experience did you have?” Q2. “How might this”trainer the trainer” influence your future GME faculty roles?” Q3. “Do you have any other comments or suggestions for the faculty who developed this ultrasound workshop series?”

### Logistics

The course was delivered at three separate sites. The medical school sponsor of the GME consortium (Michigan State University College of Osteopathic Medicine Statewide Campus System) provided space and audio-visual resources at the three separate campuses to host the courses. The live human models at each table were volunteer first or second-year medical student members of the EM student interest group at the authors’ sponsoring medical school. Two students volunteered to serve as models for instructor training. A total of ten students volunteered to cover the resident training session. Prior to their participation, each student signed a waiver form stating understanding that any possible abnormal findings discovered during the course would not be diagnosed and would require follow up with their primary care physician.

## RESULTS

The authors obtained pre- and post-test score data from a total convenience sample of N = 42 EM residents who had attended one of the three courses. Residents came from 11 different EM residency programs. Eighteen (42.8%) were PGY1 or PGY2 residents, with the remaining 23 (54.8%) residents either PGY3 or PGY4 (with one participant not specifying their PGY year). Twenty-nine (69.0%) residents described themselves as male. The three workshop site cohorts were comprised of 17, 13 and 12 resident learners. Of the total sample, one (2.3%) resident only completed a pre-course test. See Table 1 for descriptive characteristics and POCUS exam score patterns of the study sample.

**Table 1.** Descriptive Statistics of Pre-Post-Course Scores (N = 42 Emergency Medicine Residents)

	**N** **(Percent)**
**Gender**	
Male	29 (69.0%)
Female	12 (28.6)
Missing	1 (2.4)
**PGY Year**	
One	10 (23.8)
Two	8 (19.0)
Three	12 (28.6)
Four	11 (26.2)
Missing	1 (2.4)
	**Mean (SD) range**
**Number of Correct PRE-Course Exam Answers** (n = 41) (10 items, possible range 0 to 10)	6.56 (1.733) (range 3 to 10)
**Number of (Corrected) Correct POST-Course Exam Answers** (n = 41) * (20 items, possible adjusted range 0 to 10)	8.8646 (1.044) (5 to 10)
**Change in Number of Correct Pre- to Post-Course Exam Answers** (n = 40)	2.10 (1.642) (- 1.00 to + 5.50)

* Equally-weighted Correct answers in Post-course Test divided by two.

**Figure 1.**
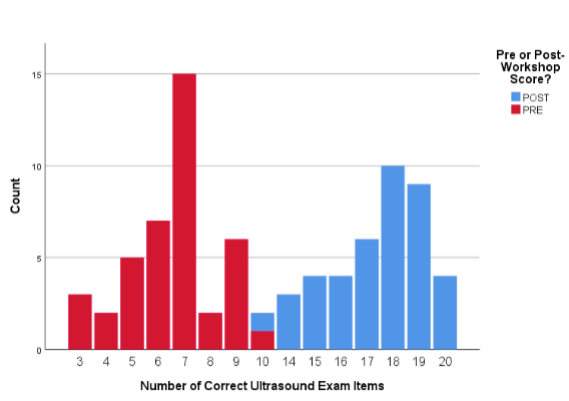
Overall Proportionate Changes in Pre-to-Post-Course Test Scores (n = 40)

As depicted in Figure 1, the scores of 35 (87.5%) of total sample resident learners’ scores increased from pre- to post-test scores, with five (11.9%) other residents maintaining their pre-course score levels. Only two (4.8%) residents experienced a pre-post score decline.

### Pre-to- post-course test score changes

A series of non-parametric Wilcoxon Matched Pairs Signed Rank Test procedures, a type of statistical test especially suitable for smaller samples in which the distribution of scores is not presumed to be *normally* distributed, was conducted.^18^ This procedure demonstrated a statistically significant two-tailed increase in number of correct pre- to post-course score differences (t = 8.091, df 39, p < 0.001). (See Table 2)

During the subsequent ANCOVA procedures, the significance of pre- post-course test score changes persisted (F2, 39) = 2.757, p = 0.039). Neither resident characteristic model terms (i.e., gender, or PGY year category) approached statistical significance (See Table 2)

**Table 2.** Potential Predictors of POCUS Pre-Post-Course Test Score Differences (N = 42 Emergency Medicine Residents)

	**Std. Error Mean**	**t**	**df**	**Sig** **(2-tailed)**
Number of Correct Pre-Course Answers - Number of Correct Post-Course Answers *	**0.25956**	**8.091**	**39**	**< 0.001**
				
	**Mean Square**	**F**	**Df**	**Sig** **(2-tailed)**
Number of Correct Pre-Workshop Answers - Number of Correct Post-Workshop Answers **				
Intercept	1637.669	1768.683	1	0.000
Difference from Pre-Workshop to Post-Workshop Exams Score	2.553	2.757	7	**0.039**
PGY Year Category	1.080	1.167	1	0.294
Gender	0.382	0.413	1	0.529
Score Difference * PGY Year	1.595	1.722	4	0.189
Score Difference * Gender	0.696	0.751	4	0.570
Score Difference * PGY Year * Gender	0.908	0.981	2	0.394

**Bold P values** significant at Alpha of p < 0.05.

* Wilcoxon Matched Pair Signed Rank T Test

** Analysis of Covariance (ANCOVA) Procedures

### Pre-to- post-course POCUS test score changes across sites

When stratifying the three learner cohorts by training sites, a separate ANCOVA model failed to show statistically significant differences in residents’ overall pre-post score improvements across the three sites (F2, 39) = 2.529, p = 0.039. This finding indicates that the overall positive impact of the course on pre-post score differences was consistent across the three training sites.

Another indication of the consistency of how the residents received the course was the quantitative evaluations data received from the three learner cohorts. Residents responded to five summary evaluation items on a Likert-type numerical scale. (“0” as least favorable and “5” as most favorable). Mean cohort evaluation scores averaged 4.756, 4.822 and 4.844 from 31 (73.8%) of the 42 residents.

### Qualitative Survey Results

**Figure 2.**
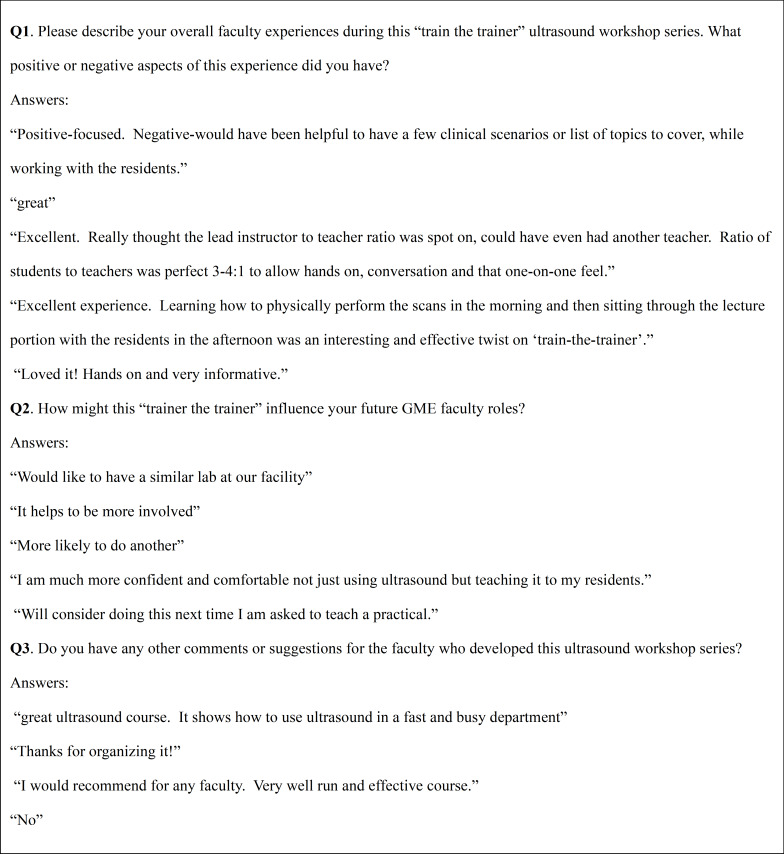
Instructor Post-Course Survey Questions and Exemplar Answers

This was the first attempt by the consortium to utilize non-paid physicians to serve as instructors for an ultrasound course. The assumption was the instructors would perceive value in free POCUS instruction, credit for scholarly activity, and five hours of 1-A CME. Several direct exemplar quotes from the qualitative survey sent to the instructors are listed in Figure 2. The survey results were overwhelmingly positive and the instructor debriefings after each course also reflected positive experiences with willingness to participate as volunteer instructors for future courses.

## DISCUSSION

The evaluation of this pilot EM POCUS course demonstrated success on several fronts. First, the ‘train the trainer’ model demonstrated an effective way to provide ultrasound training to EM residents. Educational material was communicated in a consistent way for residents and attending physicians that allowed for acquisition of skill and knowledge. This model was chosen to address two specific challenges: first, providing a course to residents who were geographically dispersed and second, training novice instructors to facilitate hands-on instruction.

Further use of the ‘train the trainer’ model would be most beneficial for programs where these issues are a challenge. It may not be as applicable to a single system residency program that is not geographically spread out where there are many with ultrasound expertise to provide training. That being said, this model could be a way to present additional training to practicing physicians who have little ultrasound experience. To this point, the education imparted to the bedside instructors, who were themselves ultrasound novices, made them more comfortable using POCUS techniques. Beyond improving their ultrasound acumen, the faculty involved collected CME as well as received scholarly activity hours. The opportunity to fulfill multiple faculty requirements provided additional benefit for the instructors.

There are several areas for future studies. Subsequent studies could consider comparing residents taught by EM instructors who have extensive ultrasound experience with those with less experience. Future studies could also determine if the timeframe for instructor training is appropriate, or if a longer timeframe for instructor training would provide even more skill acquisition and improved teaching for the instructors. Because this is a recurring course, there is also an opportunity for the results to be validated on a larger group of participants to evaluate if the positive results are consistent when applied to additional learners.

## LIMITATIONS

The results come from a smaller convenience sample of 42 EM residents from a finite number of EM residency programs. This project may have been underpowered to detect potentially significant sample subgroup differences (e.g., male vs. female learners) relative to pre- and post-workshop score differences. The authors also acknowledge that measured increases in residents’ POCUS test scores may have been skewed by a Hawthorne/observer effect since participating residents were fully aware that the authors were monitoring their test scores.

## CONCLUSION

The evaluation of this novel model supports the feasibility of the ‘train the trainer’ program. Despite our small sample size, these results show a proportionate increase in pre- to post-test scores among participating residents. Based on our results, beneficial training was provided to the residents as well as the instructors. This model provides an additional option for EM residency program educators to consider when developing their POCUS training courses. 

NOTE: The review of this manuscript was coordinated by SMRJ Assistant Editor Sam Wisniewski

### Conflict of Interest

The authors declare no conflict of interest.
